# Somatic Comorbidity in Chronic Constipation: More Data from the GECCO Study

**DOI:** 10.1155/2016/5939238

**Published:** 2016-10-16

**Authors:** Paul Enck, Johannes Leinert, Menno Smid, Thorsten Köhler, Juliane Schwille-Kiuntke

**Affiliations:** ^1^Department of Internal Medicine VI, University Hospital Tübingen, Tuebingen, Germany; ^2^Infas Institute, Bonn, Germany

## Abstract

*Background*. Comorbidity in chronic constipation has rarely been investigated, despite the fact that constipation can occur as one symptom in a number of neurological, systemic, and other nonintestinal and intestinal disorders.* Methods*. Of 1037 individuals with constipation identified during a telephone survey, 589 returned a postal questionnaire with valid data, asking for sociographic data, clinical symptoms, comorbid conditions, medication intake, and health care behavior related to constipation. Among them, 245 reported some somatic diagnoses and another 120 regular medication intake. They were compared to individuals without comorbid condition and presumed functional constipation (*n* = 215).* Results*. Individuals reporting a somatic comorbid condition and/or regular medication were significantly older than those with functional constipation (63.8 ± 15.8 and 43.7 ± 15.5 years, resp., *p* < 0.001) and had lower health and social status (both *p* < 0.001), but similar general life satisfaction (n.s.). Their quality-of-life was lower for the physical (*p* < 0.001) but not for the mental health domain (n.s.), while among those with functional constipation, the mental health domain distinguished IBS-C individuals from those with functional constipation but without pain (*p* < 0.001).* Conclusion*. In an unselected population sample with constipated individuals, those with a somatic comorbid condition outnumber those with functional constipation alone and are distinctly different with respect to age and health status.

## 1. Background

Prevalence of chronic constipation has been reported to vary between 5% and 15%, depending on the size and type of assessment, the definition of constipation, and variables such as nationality, culture, and the health care system [1–3] in which the survey is conducted. In previous papers we reported constipation to be present in 14.9% of a representative population sample from Germany during a telephone survey [4] and comorbidity to be high in those allowing further evaluation by a postal questionnaire [5]. We also noted a strong self-selection bias when stepping down from a random population sample towards in-depth evaluation of accompanying symptoms in those who acknowledge chronic constipation symptoms [5].

Constipation may occur as a secondary symptom, for example, in a number of neurological (e.g., stroke, Parkinson's disease, and spinal cord injury), systemic (diabetes, hypothyroidism, and scleroderma), and other disorders, to intestinal or nonintestinal surgery [6–8], or to a variety of medications used for treatment of chronic clinical conditions, for example, calcium antagonists for high blood pressure [9], opioids for chronic pain [10], and tricyclic antidepressants for major depression [11]. However, because of is frequency and multifactorial origin [12], constipation may also occur independent of coexisting comorbid conditions and/or medication intake. This is usually difficult to differentiate in epidemiological surveys and without physical examination [13].

However, such comorbid constipation has rarely been reported in the epidemiological literature [14], and neither has it often been compared to those with functional constipation [15]. In fact, patients with constipation and significant comorbidity are usually excluded from drug trials and presumably do profit less from new drug developments for several reasons: their efficacy is often not proven in controlled trials, the respective medical subspecialties are not or less exposed to novel drug development information, and patients and/or doctors frequently value the constipation symptoms as minor in light of the underlying neurological, systemic, or other disorders.

Among the few population-based studies addressing comorbidity in chronic constipation is a recent case-controlled study of 307 constipated individuals who were compared to an age- and sex-matched control sample [16]. It shows significant elevations of a few GI (anal surgery) and non-GI conditions (Parkinson's disease, multiple sclerosis).

The purpose of the present evaluation of the German Chronic Constipation (GECCO) study data was to describe the population with constipation with comorbidity and to compare them to a group with presumed functional constipation with respect to sociographic, clinical, health care utilization, and quality-of-life data.

We also wanted to explore the specificity of the Rome criteria for constipation-predominant irritable bowel syndrome (IBS-C) and functional constipation (FC-R) [17] to distinguish between constipation subgroups with and without somatic comorbidity.

## 2. Methods

The population prevalence data from a telephone interview with 15.000 representative adults were recently [4] reported to be 14.9%, as were the characteristics of individuals with functional constipation, that is, without any comorbid condition [5]. For further details of the methodology of GECCO, we refer to both of those previously mentioned papers [4, 5]. A questionnaire was sent to 1037 constipated individuals identified during the telephone survey who had agreed to a follow-up study.

### 2.1. The Questionnaire

The questionnaire was composed of 4 modules ((1) General Health; (2) Concurrent Diseases/Medication; (3) Health Care Utilization; and (4) Constipation and IBS); it started with some general questions regarding general health that contained the Short-Form 12 (SF-12) quality-of-life test [18]. Module 2 asked for the presence of gastrointestinal diagnoses (Crohn's disease, ulcerative colitis, GI cancer, celiac disease, and prolapse) and nonintestinal disorders (diabetes, hypothyroidism, stroke, Parkinson's disease, and multiple sclerosis) (yes, no) that are often associated with constipation and for medication intake of the most frequent drugs (generic and brands) on the German market (beta-blockers, ACE inhibitors, calcium antagonists, diuretics, statins, L-thyroxin, antidiabetics, PPI, pain medication, antidepressants, barbiturates, and sedatives), each to be answered for their frequency (daily, at least twice/week, and less). Similarly, drugs taken for constipation (macrogol, lactulose, sorbitol, bisacodyl, sodium bicarbonate, prucalopride, psyllium, Senna products, and Glauber salt) were checked for intake frequency (daily, at least twice/week, and less), efficacy, and side effects.

Module 3 asked for health care utilization: consultation of specialists in the past 12 months, sick-days because of constipation, inpatient treatment, diagnostic procedures performed, and complementary and alternative remedies taken because of CC, including the amounts spend that were not reimbursed by health insurance plans.

Module 4 contained questions from the validated German version of the Rome III modular questionnaire for IBS and for functional constipation [19].

The protocol of the study methodology had been reviewed by the Ethics Committee of the Tübingen University Medical School.

### 2.2. Statistics

Constipation subsamples were constructed based on predefined criteria: patients with at least one somatic diagnosis in addition to constipation symptoms were labelled “comorbid constipation”; if they reported regular medication intake (>twice/week) but no firm diagnoses, this group was called “presumed comorbid constipation.” The remaining (no diagnoses, no medication) were called “functional constipation” (FC). These subgroups were compared by parametric (ANOVA) and nonparametric tests (Chi-square test) where appropriate.

In order to test the specificity of the Rome criteria [19], all patients also were subdivided into IBS-C, FCR, and FC. They were labelled IBS-C when they met the following criteria: abdominal pain/discomfort at least 3 days per months in addition to constipation, for more than 6 months, not associated with the menstrual cycle, and at least 2 of the following symptoms: symptom improvement with defecation, onset associated with a change in stool frequency, and onset associated with a change in stool form; their occurrence frequency had to be often or more. The definition for FC-R [17] requires not classifying for IBS-C, and at least 2 of six symptoms (need for straining, lumpy or hard stools, sensation of incomplete evacuation, sensation of anorectal obstruction, and need for manual maneuvers to facilitate defecation more than occasionally) or less than 3 defecations/week. All remaining individuals were labelled FC.

All data are reported as mean ± SD and are unweighted with respect to the initial representative survey. The significance level was set to 0.05 for all tests.* Post hoc t*-tests and Chi-square test of subgroup comparisons were not corrected for multiple comparisons but instead only performed when the main (ANOVA, Chi-square test) analysis yielded significance.

## 3. Results

Among all 589 (56.8%) respondents of 1037 individuals approached who returned the questionnaire and provided useable data, 9 women reported being pregnant—they were excluded, leaving 580 complete data sets to be entered into this analysis.

### 3.1. Comorbid Constipation

When asked for concurrent GI and non-GI diagnoses, 245 persons (42.2%) reported one or more diagnoses to be present; these were labelled “comorbid constipation.” In 315 cases medication was taken at least twice per week; when those with a firm diagnosis were excluded, this resulted in another 120 cases with presumed comorbidity (20.7%). The data of these two subsamples are reported here. The 215 remaining cases of “functional constipation” (37.1%) serve as control sample.

### 3.2. Comorbid Constipation versus Functional Constipation

The gender distribution was similar in all groups (Table 2). Participants with comorbid and presumed comorbid constipation were significantly older (63.8 ± 15.8 and 60.6 ± 15.3 years, resp.) than individuals with functional constipation (43.7 ± 15.5 years) (*p* < 0.001). Associated with the higher age, individuals with comorbid constipation were more frequently retired and had a lower family income and an overall less satisfying health situation, but a similar general life satisfaction than the two other groups.

When asked for their acute health problems, cardiovascular and urological dominated in the comorbid constipation group compared to the functionally constipated. As shown in Table 2, significant differences were also found for the duration of constipation, doctor visits for constipation during the last 12 months, and medication intake for constipation (all highest in comorbid constipation). In most but not all cases, individuals with presumed comorbidity were in between the two other groups and in some aspects closer to the comorbid group (age, general health problems) and in others closer to the functionally constipated (especially with respect to the type and severity of constipation symptoms).

Current medication intake for constipation is highest in comorbid constipation (43.7%) and significantly lower functional constipation (23.3%) (Table 2), and among the drugs taken for constipation the following were listed: psyllium (*n* = 27), macrogols (*n* = 25), and lactulose (*n* = 18). Traditional laxatives (bisacodyl, sodium picosulfate, Senna products, and Glauber salt) were only used occasionally by individuals with comorbid constipation. If medication is taken, it appears to help the majority of individuals, and reported side effects were equally present in both groups. Among the side effects listed most are bloating (*n* = 43), abdominal pain (36), and diarrhea (*n* = 19). Other side effects (itching, skin rashes, nausea, and vertigo) are listed only occasionally by a few constipated individuals with comorbidity. Complementary and alternative medicines (CAM) (homeopathy, acupuncture, and Chinese herbal medicines) were used by an equal (small) number of the constipated in all groups. A majority of individuals in all groups claimed to have changed diet to counteract constipation, and the dietary actions include all measures listed in the questionnaire (more vegetables, more legumes, liquid intake, probiotics, etc.).

### 3.3. Specificity of the Rome Criteria

**Figure 1:**
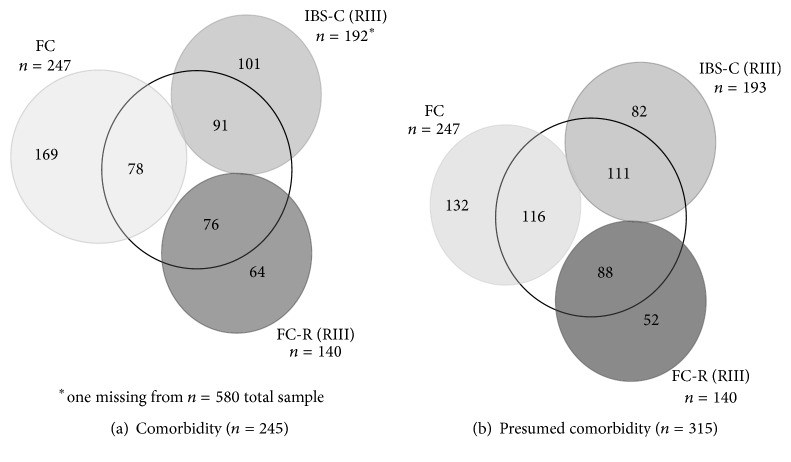
Constipated individuals matching Rome III criteria for IBS (IBS-C: *n* = 193) or for functional constipation (FC-R, *n* = 140), or not (FC: *n* = 247). (a) With acknowledged somatic comorbidity (*n* = 245, inner circle); (b) with presumed comorbid condition (*n* = 315, inner circle). Note that nearly half of individuals matching diagnostic criteria demonstrated either somatic comorbidity or presumed somatic comorbidity.

Applying the Rome III criteria to the constipated individuals with and without comorbidity, *n* = 193 individuals classified as IBS-C, *n* = 140 as FC-R, and *n* = 247 as FC. Of these, *n* = 91, *n* = 76, and *n* = 78, respectively, reported comorbid somatic disorders (Figure 1(a)). The identification based on the Rome criteria alone would thus yield a specificity of only 52.6% for IBS-C and 45.7% for FC-R. This specificity would further drop when applied to the presumed comorbid constipation group (Figure 1(b)).

Sensitivity cannot be determined since the data do not provide a gold standard for the proper diagnoses. Excluding all individuals with comorbid or presumed comorbid conditions (*n* = 365)* a priori* on the other hand (as we did in a recent paper on functional constipation [5]) would result in missing out around 50% of individuals (*n* = 91 meeting IBS-C criteria and *n* = 76 meeting FC-R criteria, *n* = 116 with FC) in whom functional constipation may be present.

Among the somatic comorbidity conditions reported by IBS-C, FC-R, and FC patients are many conditions that may be directly responsible for the constipation symptoms, as is evidenced in Table 1. Compared to population prevalence of the respective diseases, more individuals than expected with nongastrointestinal disorders (hypothyroidism, stroke, scleroderma, Parkinson's disease, and multiple sclerosis) and gastrointestinal diagnoses (inflammatory bowel diseases) were found in our cohort of constipated patients.

However, whether or not these diseases are responsible for the constipation or whether they are co-occurring and coincidental is nothing one can determine by questioning alone but requires thorough clinical evaluation.

### 3.4. Quality of Life

**Figure 2:**
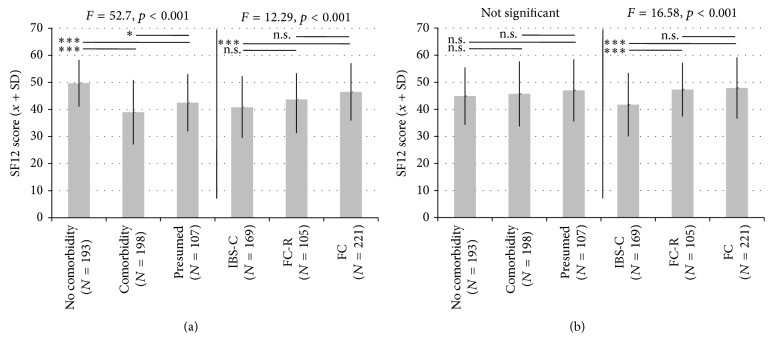
SF-12 quality-of-life measure in individuals with constipation. (a) In the SF12 physical domain; left panel: individuals with acknowledged or with presumed somatic comorbidity or without comorbidity; right panel: in individuals matching IBS-C, FC-R, and FC criteria (see text for details). (b) In the mental domain. In each panel, *F*-values indicate significance in the between-group ANOVA; “*∗*” indicates significance in* post hoc t*-tests (uncorrected): ^*∗∗∗*^ *p* < 0.001 and ^*∗*^ *p* < 0.05.

Individuals with comorbid or presumed constipation had significantly (both *p* < 0.001) lower QOL on the SF-12 physical health domain in comparison to the group with no comorbidity. No difference was found with respect to the mental health domain (Figure 2(a)). When the QoL was evaluated age-adjusted, differences were still significant for the physical and not for the mental domain (*p* < 0.001).

When QoL was compared between the IBS-C, FC-R, and FC groups irrespective of comorbidity, both the physical and the mental domain SF12 subscales were significantly lowered in IBS-C as compared to the FC groups (Figure 2(b)), also following age adjustment. However, only the mental scale also significantly distinguished IBS-C from FC-R and FC, while FC-R and FC were not different on either scale.

## 4. Discussion 

In a first GECCO report, we found 14.9% of 15.000 representative German adults to be constipated [4]. This perfectly matches what has been reported across 45 European and non-European countries [12] but corrects the data from a previous study with smaller numbers [20]. In a subsequent analysis [5] we found individuals with functional constipation (IBS-C, FC-R, and FC) to be similar with respect to most of the social and clinical descriptors assessed in our survey. When this group as a whole was compared to a group with acclaimed or presumed comorbidity in the present report, individuals with “somatic constipation” were significantly older than those with functional constipation and had lower health and social status, but similar general life satisfaction. Their quality-of-life was lower for the physical but not for the mental health domain, while among those with functional constipation, the mental health domain distinguished IBS-C individuals from those with functional constipation but without pain. The later finding has also been found in previous analyses, for example, [21–23].

Among the 45 studies listed by Suares and Ford [12] with global epidemiological data on constipation, only a few reported the prevalence of somatic and/or psychiatric comorbidity; on the other hand, constipation is frequently reported as a comorbid condition of other diseases such as stroke, Parkinson's disease, spinal cord injury, multiple sclerosis, scleroderma, and diabetes [6, 8, 24, 25]. As we can show here, the self-selection bias that we have described in our questionnaire survey [5] may be specifically due to individuals that suffer from constipation associated with (but not necessarily due to) a number somatic diseases, both with a high general population prevalence, such as hypothyroidism and stroke, and with low prevalence rates such as scleroderma and inflammatory bowel diseases. Because of this comorbidity, but presumably also because of the overall higher age, individuals with comorbid conditions perceive their constipation as more severe and do utilize the health care system more frequently than constipated without comorbidity.

Systematic exploration of somatic comorbidity in chronic constipation has become a topic in research only recently, but an early discussion was initiated by Talley et al. in 2003 [26]. Among more than 7,000 chronically constipated patients in a sample from private practice and collected over a period of 10 years, neurological diseases (Parkinson's disease, multiple sclerosis) accounted for 3.2 and 0.7%, respectively, of cases, similar to diabetes (4.7%) and thyroid disease (5.9%). Opioids, diuretics, antidepressants, antispasmodics, anticonvulsants, and aluminum antacids were all associated with higher risk of constipation than in controls. To recognize this in clinical routine is of specific importance, as it may guide patient management both with respect to the underlying disorder and with respect to the constipation symptoms.

Nellessen et al. [15] reviewed the literature for intestinal and nonintestinal symptoms associated with chronic constipation (and with IBS-C), showing that in 35 studies of different type and sample size, increased incidences of depression, overweight, obesity, and diabetes were reported to be associated with constipation. Mody et al. [14] identified 28,000 individuals with chronic constipation in a US health insurance database and noted increased rates of depression and mood disorders (14.2%), hypothyroidism (9.8%), and other neurological disorders (9.7%), as compared to nonconstipated controls.

Rey et al. [22] compared constipated patients with and without abdominal pain to IBS-C patients, recruited from the Spanish population, but did not report comorbid somatic conditions, presumably due to the fact that this study was based on a telephone survey, similar to our study [4], that did not allow extensive questioning. Choung et al. [16] finally compared a more than 300 chronically constipated to a control sample of equal size and found—in a questionnaire survey—a significantly increased prevalence of Parkinson's disease (4%), while most other conditions (including all GI diseases) were not different to a matched control cohort. Metabolic disorders and other neurological diseases were moderately elevated in constipation (*p* < 0.10). When all cases of chronic constipation were compared to the complete control sample (>2,000 subjects), the prevalence of multiple sclerosis, other neurological diseases, metabolic diseases, hypothyroidism, cardiovascular diseases, and psychiatric diseases all reached significance levels. While we are missing a nonconstipated control cohort in our study, our data match those reported by Choung et al. [16]. Unfortunately, our questionnaire survey did not include a psychometric tool to assess psychiatric comorbidity, as this has been reported to be elevated in constipations as well [15, 16]; however, in this case, constipation may likely be the consequence of antidepressant medication and has not been documented in most of these studies, except by Talley et al. [26].

Decreased quality-of-life has been frequently documented in constipation [1, 23, 27–37] and has been documented for our cohort as well [5], but comparison of different constipation subsamples is rare: here we show that, for the physical domain of the SF12, comorbidity (as well as presumably the increased age of those with comorbid constipation) differentiates better than the IBS classification, while, for the mental domain, comorbidity does not appear to play a role at all, in contrast to the presence/absence of pain that does. The latter has also been noted in other studies [22]. Pain is the major factor responsible for lower quality of life in all functional bowel disorders [23].

The validity of the Rome criteria [17] for functional bowel disorders, especially for IBS-C, has been tested [38, 39] as well as questioned [40, 41] ever since their first appearance [42]. However, their specificity in a cohort of constipated with or without comorbidity is reported here for the first time: nearly 50% of individuals that match IBS-C criteria report comorbidity that is incompatible with a diagnosis of a functional bowel disorders, and among them there are many that regularly take medication indicative of a chronic gastrointestinal or extraintestinal condition. Unfortunately, an epidemiological survey such as ours cannot verify the accuracy of these self-reported diagnoses and thus will always require confirmation by a case-controlled clinical study.

Besides the limitations already discussed above, more limitations of our data analysis need acknowledgment. We also used rather liberal criteria to define “comorbid constipation” based on self-reported diagnoses and/or regular medication intake, the latter with a cut-off of 2 or more days per week. This may have inflated the number of individuals that were assembled in the group with “comorbid constipation” and downsized the number of patients with functional constipation for this analysis, since regular use of a PPI does not necessarily imply functional dyspepsia or gastric ulcer or GERD. In some cases individuals reported intake of diabetic medication but not the diagnosis of diabetes, which may shed light on the comprehension of the questionnaire by some participants. Finally, the presence of a somatic disease not necessarily indicates that constipation is caused by this disease; it may be incidental comorbidity, and the absence of a somatic condition in those labelled functional constipation does not confirm that a comorbid somatic condition does not exists in these cases; epidemiological data relying solely on subjective reports always carry the risk of false information. Therefore, some of the volunteers labelled as “comorbid constipation” and excluded for this analysis may instead belong to one of the groups included, and such correction may diminish some of the found differences, although the opposite may happen as well.

## Figures and Tables

**Table 1:** Somatic diagnoses reported by constipated individuals identified as meeting IBS-C, FC-R, and FC definitions (number of cases, more than one allowed). Bold indicates increased prevalence as compared to population data.

Self-reported diagnoses	IBS-C	FC-R	FC	All	P^*∗*^ (%)	PP^#^ (%)
*N*	91	76	78	245 + 335 = 580		
	*Non-GI diagnoses*					
Diabetes	10	23	19	52	8.9	10.0
Hypothyroidism	38	34	35	107	**18.4**	10.0
Stroke	10	7	12	29	**5.0**	1.0
Scleroderma	1	2	0	3	**0.5**	0.01
Parkinson's disease	1	4	0	5	**0.8**	0.25
Multiple sclerosis	4	2	0	6	**1.0**	0.15

	*GI diagnoses*					
Crohn's disease	1	1	2	4	**0.7**	0.04
Ulcerative colitis	6	3	2	11	**1.9**	0.08
Celiac disease	2	0	0	2	0.3	1.0
GI cancer	19	19	19	57	9.8	???
Anal stenosis	23	12	2	37	6.4	???
Anal/rectal prolapse	13	5	5	23	3.9	???

^*∗*^Prevalence (%) in the combined cohort with and without comorbidities.

^#^Population prevalence (%) of the disease according to crude epidemiological data.

**Table 2:** Sociographic data, health problems, and life satisfaction in comorbid constipation (*n* = 245) as compared to constipated individuals with regular medication (presumed comorbidity) (*n* = 120) and without comorbid condition (functional constipation) (*n* = 215).

Variable name	C with comorbidity	C with presumed comorbidity	Functional constipation	Statistics^#^	*Post hoc* tests		
*N*	245	120	215		1-2	1–3	2-3
Age (mean, SD)	63.8 ± 15.8	60.6 ± 15.3	43.7 ± 15.5	*p* < 0.001	n.s.	*∗∗*	*∗∗*
Male : female	93 : 152	37 : 83	71 : 144	n.s.	—	—	—
Height (m)	1.68 ± 0.09	1.68 ± 0.83	1.69 ± 0.93	n.s.	—	—	—
Weight (kg)	77.3 ± 16.4	76.0 ± 18.4	71.0 ± 15.6	*p* < 0.001	n.s.	*∗*	*∗∗*
BMI	27.2 ± 5.2	26.9 + 6.0	24.5 ± 4.7	*p* < 0.001	n.s.	*∗∗*	*∗∗*

*Sociographic data*							
Education: secondary^+^	50	28	75	*p* < 0.001	n.s.	*∗∗∗*	*∗∗*
Full-time/part time (1)	56	22	112	*p* < 0.001	*∗*	*∗∗∗*	*∗∗∗*
Mini job, occasional (2)	7	13	26				
Not working, training (3)	2	4	3				
Parent time (4)	1	3	11				
Retired (5)	149	63	27				
Income: >2,500 €/mo	45	31	81	*p* < 0.001	n.s.	*∗∗∗*	n.s.

*Overall life satisfaction*							
Fully (1)	66	48	69	n.s.	—	—	—
Rather (2)	133	57	117				
Rather not (3)	30	11	23				
Not at all (4)	15	4	6				

*Overall general health*							
Very good (1)	8	6	48	*p* < 0.001	n.s.	*∗∗∗*	*∗∗∗*
Good (2)	71	42	99				
Satisfactory (3)	90	35	41				
Less good (4)	48	23	20				
Bad (5)	27	14	6				

*Health problems*							
Sick the last 4 wks: no	150	80	172	*p* = 0.008	n.s.	*∗∗∗*	n.s.
Back pain: yes	177	84	125	*p* = 0.004	n.s.	*∗∗*	*∗*
Circulation: yes	115	52	53	*p* < 0.001	n.s.	*∗∗∗*	*∗∗*
Gynaecological: yes	8	13	21	n.s.	—	—	—
Urological: yes	56	13	13	*p* < 0.001	*∗∗*	*∗∗∗*	n.s.
Gastrointestinal: yes	96	32	61	n.s.	—	—	—

*Constipation symptoms*							
Duration (years)	12.9 ± 17.9	13.1 ± 15.7	8.9 ± 10.8		*∗∗*	*∗*	*∗*
4-week prevalence	131	40	62	*p* < 0.001	*∗∗∗*	*∗∗∗*	n.s.
To doctor	74	26	31	*p* = 0.001	n.s.	*∗∗∗*	n.s.
Medication	107	37	50	*p* < 0.001	*∗*	*∗∗∗*	n.s.
<3 stools/week	83	34	79	n.s.	—	—	—
Straining	167	77	135	n.s.	—	—	—
Hard stools	188	82	161	n.s.	—	—	—

^#^ANOVA: univariate, 3 groups, or Chi-Square: in case of significance, pairwise *post hoc* comparisons; ^+^number with secondary school finished (maturation); ^*∗∗∗*^ *post hoc* testing: ^*∗∗∗*^ *p* < 0.001; ^*∗∗*^ *p* < 0.01; ^*∗*^ *p* < 0.05; and n.s.: not significant.
